# Identification of runs of homozygosity associated with male fertility in Italian Brown Swiss cattle

**DOI:** 10.3389/fgene.2023.1227310

**Published:** 2023-07-06

**Authors:** Hendyel A. Pacheco, Attilio Rossoni, Alessio Cecchinato, Francisco Peñagaricano

**Affiliations:** ^1^ Department of Animal and Dairy Sciences, University of Wisconsin-Madison, Madison, WI, United States; ^2^ Italian Brown Breeders Association, Verona, Italy; ^3^ Department of Agronomy, Food, Natural Resources, Animals and Environment, University of Padova, Padua, Italy

**Keywords:** inbreeding depression, homozygosity, service sire fertility, genetic diversity, dairy cattle

## Abstract

Intensive selection for improved productivity has been accompanied by an increase in inbreeding rates and a reduction in genetic diversity. The increase in inbreeding tends to impact performance, especially fitness-related traits such as male fertility. Inbreeding can be monitored using runs of homozygosity (ROH), defined as contiguous lengths of homozygous genotypes observed in an individual’s chromosome. The goal of this study was to evaluate the presence of ROH in Italian Brown Swiss cattle and assess its association with bull fertility. First, we evaluated the association between ROH and male fertility using 1,102 Italian Brown Swiss bulls with sire conception rate records and 572 K SNPs spanning the entire genome. Second, we split the entire population into 100 high-fertility and 100 low-fertility bulls to investigate the potential enrichment of ROH segments in the low-fertility group. Finally, we mapped the significant ROH regions to the bovine genome to identify candidate genes associated with sperm biology and male fertility. Notably, there was a negative association between bull fertility and the amount of homozygosity. Four different ROH regions located in chromosomes 6, 10, 11, and 24 were significantly overrepresented in low-fertility bulls (Fisher’s exact test, *p*-value <0.01). Remarkably, these four genomic regions harbor many genes such as *WDR19*, *RPL9*, *LIAS*, *UBE2K*, *DPF3*, *5S-rRNA*, *7SK*, *U6*, and *WDR7* that are related to sperm biology and male fertility. Overall, our findings suggest that inbreeding and increased homozygosity have a negative impact on male fertility in Italian Brown Swiss cattle. The quantification of ROH can contribute to minimizing the inbreeding rate and avoid its negative effect on fitness-related traits, such as male fertility.

## 1 Introduction

Dairy cattle breeding programs currently face the challenge of achieving rapid genetic progress while maintaining adequate genetic diversity and low inbreeding levels. The intense selection for high-producing dairy cows commonly results in the widespread use of related individuals as parents of the next generation. Such selection practice leads to a decrease in the effective population size, a reduction in genetic diversity, and an increase in inbreeding levels. The major concern is that high levels of inbreeding can cause a reduction in fitness and overall performance, a phenomenon commonly known as inbreeding depression (Falconer and Mackay, 1996). Specifically, fitness traits such as survival, reproduction, and disease resistance are more affected by inbreeding depression. Managing inbreeding levels and maintaining genetic diversity is crucial for the long-term health and productivity of dairy cattle populations.

The adverse effects of inbreeding negatively impact many economically relevant traits in dairy cattle. Some examples are male and female fertility (Dorado et al., 2017; Ferencakovic et al., 2017; Nani and Peñagaricano, 2020), survival (Thompson et al., 2000a; Thompson et al., 2000b; McParland et al., 2007), and even milk production (McParland et al., 2007; Bjelland et al., 2013; Pryce et al., 2014; Howard et al., 2015; Makanjuola et al., 2021). It is noteworthy that the degree of inbreeding depression may vary across traits due to differences in dominance effects and epistasis (Doekes et al., 2021).

Traditionally, inbreeding depression is estimated as a regression of the phenotype of interest on pedigree-based inbreeding coefficients (Curik et al., 2014). The inbreeding coefficient (F) is the probability that two genes at any locus in an individual are identical by descent (IBD) (Falconer and Mackay, 1996). However, there are some limitations to calculating inbreeding coefficients using pedigree information. For instance, with the pedigree-based method, the inbreeding coefficient represents the expected fraction of the genome that is IBD, and the accuracy of this estimation relies on the accuracy of the genealogical data (McQuillan et al., 2008; Keller et al., 2011). Moreover, the pedigree-based method does not capture the variation due to Mendelian sampling and linkage during gamete formation (Hill and Weir, 2011). Fortunately, with the increasing availability of genomic information, more precise estimates of inbreeding coefficients can be obtained using genotypic data. Indeed, single nucleotide polymorphisms (SNPs) offer new opportunities to improve the accuracy of inbreeding coefficient estimation and develop more detailed approaches for detecting inbreeding depression (McQuillan et al., 2008; Keller et al., 2011). Genomic inbreeding coefficients can be estimated from the diagonal elements of the genomic relationship matrix calculated from SNP data (VanRaden, 2008; Maltecca et al., 2019). One approach to obtain these genomic inbreeding estimates is to use an SNP-by-SNP method (Keller et al., 2011; Marras et al., 2015). Nonetheless, this method does not distinguish alleles that are IBD and identical by state (IBS), resulting in an overestimation of the inbreeding coefficients.

Another approach, involving runs of homozygosity (ROH), has been proposed to accurately capture alleles that are IBD using genomic information. This method relies on the determination of autozygous segments based on runs of consecutive homozygous genotypes, assumed to evolve from a common ancestor (McQuillan et al., 2008). Based on the length, ROH are divided into short, which indicates distant inbreeding, and long, which indicates recent inbreeding (Kirin et al., 2010; Ferencakovic et al., 2013; Curik et al., 2014). Identifying and characterizing ROH can provide an insight into how population history, structure, and demography have evolved (Peripolli et al., 2017). In fact, the use of ROH is a powerful tool to aid the designing of mating systems to minimize inbreeding rates. ROH metrics allow for identifying specific genomic regions linked to inbreeding depression and for revealing regions under strong selection (Bjelland et al., 2013; Kim et al., 2013).

The impact of inbreeding and increased homozygosity on fitness traits, such as cow fertility, has been studied in dairy cows. However, the impact of inbreeding and increased homozygosity on dairy bull fertility has been largely overlooked. Interestingly, there is a substantial variation in the conception rate among Italian Brown Swiss bulls, with a more than 20% conception rate difference between high-fertility and low-fertility bulls (Pacheco et al., 2021). The main goal of this study was to investigate the association between homozygosity and male fertility in the Italian Brown Swiss cattle population. We evaluated the presence of ROH and assessed its association with the sire conception rate.

## 2 Material and methods

### 2.1 Phenotypic data

Phenotypic data consisted of sire conception rate (SCR) records from a total of 1,102 Italian Brown Swiss bulls. The sire conception rate is a phenotypic evaluation calculated using cow field data (Pacheco et al., 2021). Briefly, we first evaluated cow pregnancy success (a binary trait; 0 = failure, 1 = pregnancy) using factors related to both the bull under evaluation and the cow that receives the dose of semen. Factors that were related to the cow included lactation number, age, days in milk at breeding, and total milk yield as fixed effects. The herd-year-season and both cow additive genetic and permanent environmental effects were utilized as random effects. We then estimated the sire conception rate using only the factors related to the service bull. The factors related to the bull included age and inbreeding as fixed effects and AI company and service sire as random effects. The estimates of the sire conception rate were calculated as deviations from the mean, which was set at 0, such that each difference of +1 point reflects a 1% increase in the conception rate, while a difference of −1 point reflects a 1% decrease. For bulls with multiple SCR records, the most reliable SCR record, i.e., the SCR record with the most breeding, was used in this study. The sire conception rate values ranged from −22.3% to 9.9%, and the number of breeding ranged from 50 to 8,110.

### 2.2 Genotypic data

The genotypic data for 572,528 single nucleotide polymorphism (SNP) markers located in autosomal chromosomes were available for all the 1,102 Italian Brown Swiss bulls with SCR records. This set of SNP markers is a subset of the markers available in the BovineHD Genotyping BeadChip (Illumina Inc., CA, United States). The SNP information, which included the chromosome and position, was based on the bovine reference genome ARS-UCD-1.2.

### 2.3 Discovery of ROH

The software PLINK (version 1.9) was used to detect segments of consecutive homozygous SNPs called runs of homozygosity (ROH) (Purcell et al., 2007; Chang et al., 2015). Briefly, the algorithm in PLINK takes a window of a pre-defined number of SNPs and slides this window across the entire genome. At each window, the software determines whether this window is homozygous (yes or no). Then, for each SNP, it calculates the proportion of homozygous windows that overlap that position. Here, ROH were discovered using a sliding window of 50 SNPs, allowing one possible heterozygous genotype (to account for potential errors in genotyping) and one missing SNP per window, with a maximum gap of 500 kb between consecutive homozygous SNPs. The following PLINK parameters were used: --homozyg-window-snp 50 --homozyg-window-het 1 --homozyg-window-missing 1 --homozyg-gap 500. The rest of the parameters were used as the software default. Note that SNPs were not pruned for low minor allele frequency and/or linkage disequilibrium. These settings were selected based on the guidelines suggested by Meyermans et al. (2020). The size of the window was chosen to avoid detecting ROH segments that were IBS but not IBD (Martikainen et al., 2017).

### 2.4 Characterization of ROH

The total number of ROH segments, the average and maximum length in kilobases, and the total number of SNPs per ROH were calculated for all the animals for the autosomal chromosomes. For each chromosome, the proportion of homozygous regions was calculated as the total ROH length (calculated as the sum of all ROH segments in kb) divided by the chromosome length to explore differences in homozygosity between the chromosomes. The Pearson’s correlation coefficient between ROH and pedigree inbreeding was also calculated. The pedigree inbreeding was estimated using a pedigree file with more than five generations (Pacheco et al., 2021). The potential association between homozygosity (ROH) and male fertility (SCR) was assessed by regressing the SCR records on either the total length of ROH in Mb or the total number of ROH segments using a simple linear regression model.

### 2.5 Comparison of ROH

To investigate the differences in homozygosity between low-fertility and high-fertility bulls, the entire Italian Brown Swiss bull population was split into two subsets with extreme phenotypes: the bottom 100 bulls (low-fertility bulls) and the top 100 bulls (high-fertility bulls) based on the SCR distribution. The average SCR (standard deviation) for the low-fertility and high-fertility groups was −11.6% (3.7) and 5.9% (1.5), respectively. The amount of homozygosity, calculated as the total ROH length, was compared between fertility groups.

### 2.6 Enrichment of ROH in low-fertility bulls

The genomic regions across the bovine genome where the ROH overlapped across individuals were revealed using the PLINK parameter --homozyg-group. The goal was to determine if there was an enrichment of ROH segments in the group of low-fertility bulls. For each overlapping ROH, a one-tailed Fisher’s exact test using a 2 × 2 table was performed to determine if there was a significant enrichment of this ROH region in the low-fertility group. All significant ROH regions (*p*-value ≤0.001) were mapped to the bovine reference genome ARS-UCD-1.2 to identify candidate genes associated with male fertility. The Bioconductor biomaRt R package was used to retrieve the list of genes within each genomic region of interest (Durinck et al., 2005).

### 2.7 Validation of significant ROH regions

The most significant ROH regions identified in the previous step, i.e., ROH that were significantly enriched in low-fertility bulls, were validated in the entire Italian Brown Swiss bull population (1,102 bulls) using the following linear mixed model:

y=Xb+Zu+e,

where y is the vector of phenotypic records (SCR values); b is the vector of fixed effects that includes each of the significant ROH regions as a binary trait (presence/absence); X and Z are the design matrices relating SCR records to fixed and random effects, respectively; u is the vector of animal genetic effects; and e is the vector of random residuals. The random effects u and e were distributed as $u∼N\left(0,G\sigma_{g}^{2}\right)$ and $e∼N\left(0,R\sigma_{e}^{2}\right)$ , where $\sigma_{g}^{2}$ and $\sigma_{e}^{2}$ are the additive genetic and residual variances, respectively, G is the additive genomic relationship matrix, and R is an identity matrix. The association of each ROH region with SCR was evaluated using the *t*-test, and ROH with |t-value| ≥2 were declared to be significantly associated with the sire conception rate and therefore considered as validated. This analysis was performed using the BLUPF90 family of programs (Misztal et al., 2018).

## 3 Results

### 3.1 Discovery and characterization of ROH

**FIGURE 1:**
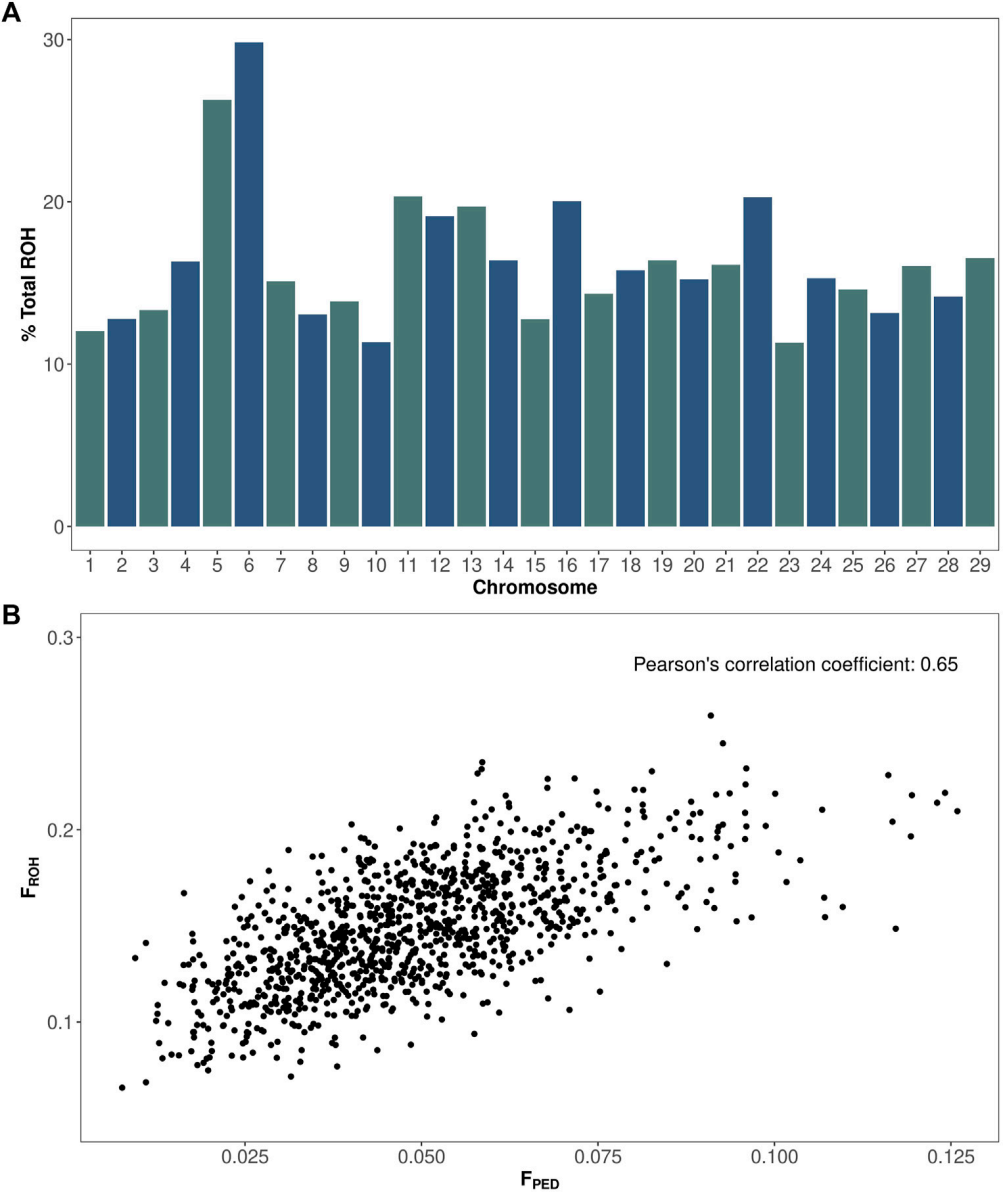
Homozygosity and inbreeding in the Italian Brown Swiss bull population. **(A)** Percentage of total homozygosity (*y*-axis) for each chromosome (*x*-axis). **(B)** ROH vs. pedigree-based inbreeding coefficients.

A total of 87,488 runs of homozygosity (ROH) were identified in the Italian Brown Swiss bull population (1,102 animals). The mean ROH length was 4,672 kilobases, equivalent to 1,038 consecutive homozygous SNPs. The ROH length ranged from 1,000 to 88,219 kilobases, and the number of SNPs ranged from 100 to 18,277. The number of ROH segments per bull ranged from 47 to 108, with an average of 79. The percentage of homozygous regions for each chromosome is shown in Figure 1A. The autosomal genome presented an average homozygosity of 16%. Chromosomes 1, 10, 15, and 23 showed the lowest degree of homozygosity, between 11% and 13%. By contrast, chromosomes 5 and 6 showed the highest level of homozygosity, at 26% and 30%, respectively. The Pearson’s correlation coefficient between ROH and pedigree inbreeding was equal to 0.65 (Figure 1B).

**FIGURE 2:**
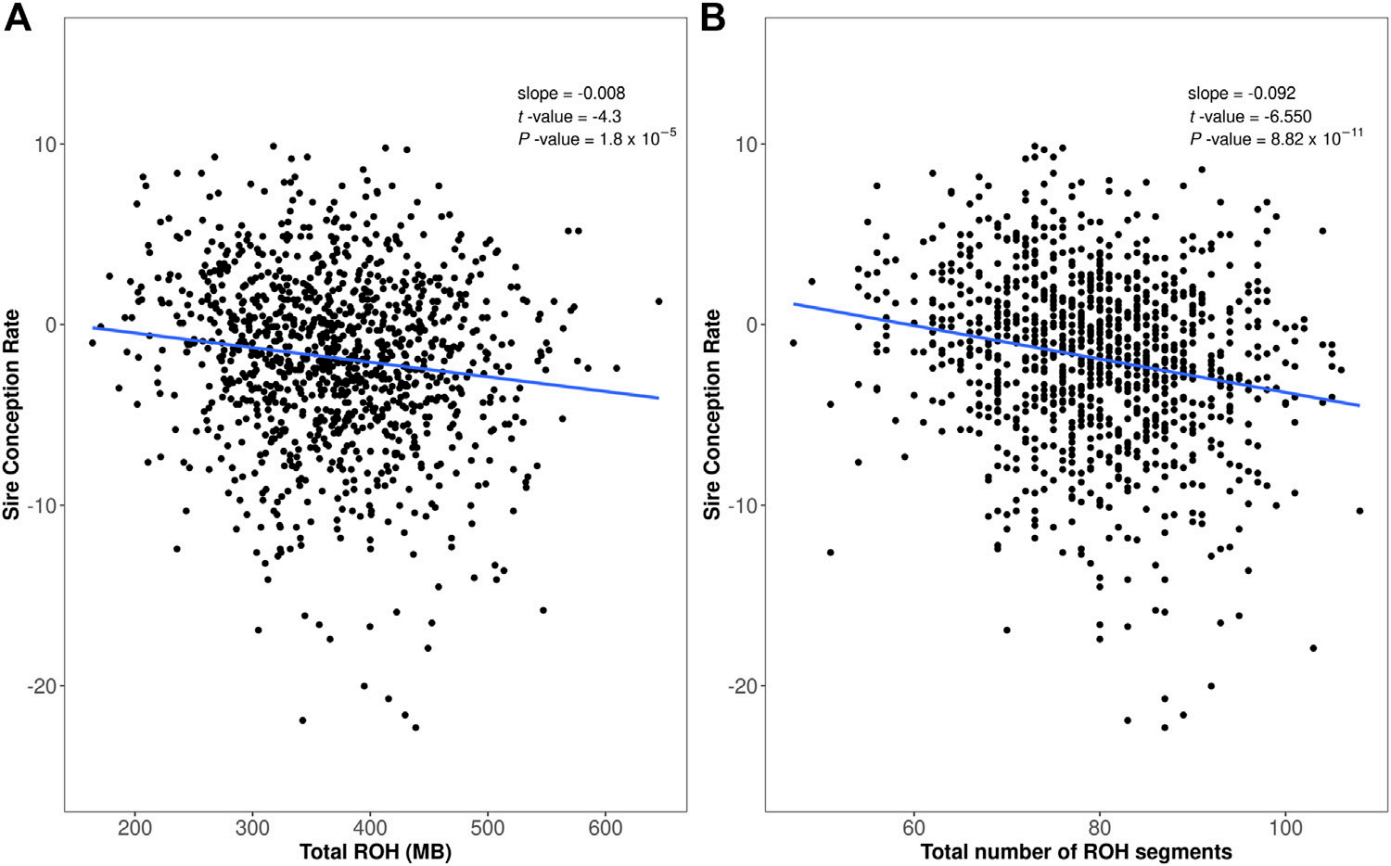
Assessment of runs of homozygosity (ROH) in the Italian Brown Swiss bull population. **(A)** Total homozygosity, calculated as total ROH length (*x*-axis), versus sire conception rate records (*y*-axis). **(B)** Total number of ROH segments per bull (*x*-axis) versus sire conception rate records (*y*-axis).

We evaluated the relationship between homozygosity and bull fertility in two ways. First, we evaluated the total homozygous regions per animal (total ROH length in Mb) versus the sire conception rate. The linear regression of the sire conception rate on ROH revealed a negative association between bull fertility and the amount of homozygosity (regression coefficient *β* = −0.008, t-value = −4.3, *p*-value ≤0.01, Figure 2A). Second, we evaluated the number of ROH segments per bull versus sire conception rate. This analysis again revealed a negative association between bull fertility and the number of ROH segments (regression coefficient *β* = −0.092, *t*-value = −6.5, *p*-value ≤0.01, Figure 2B).

### 3.2 Comparison of ROH

**FIGURE 3:**
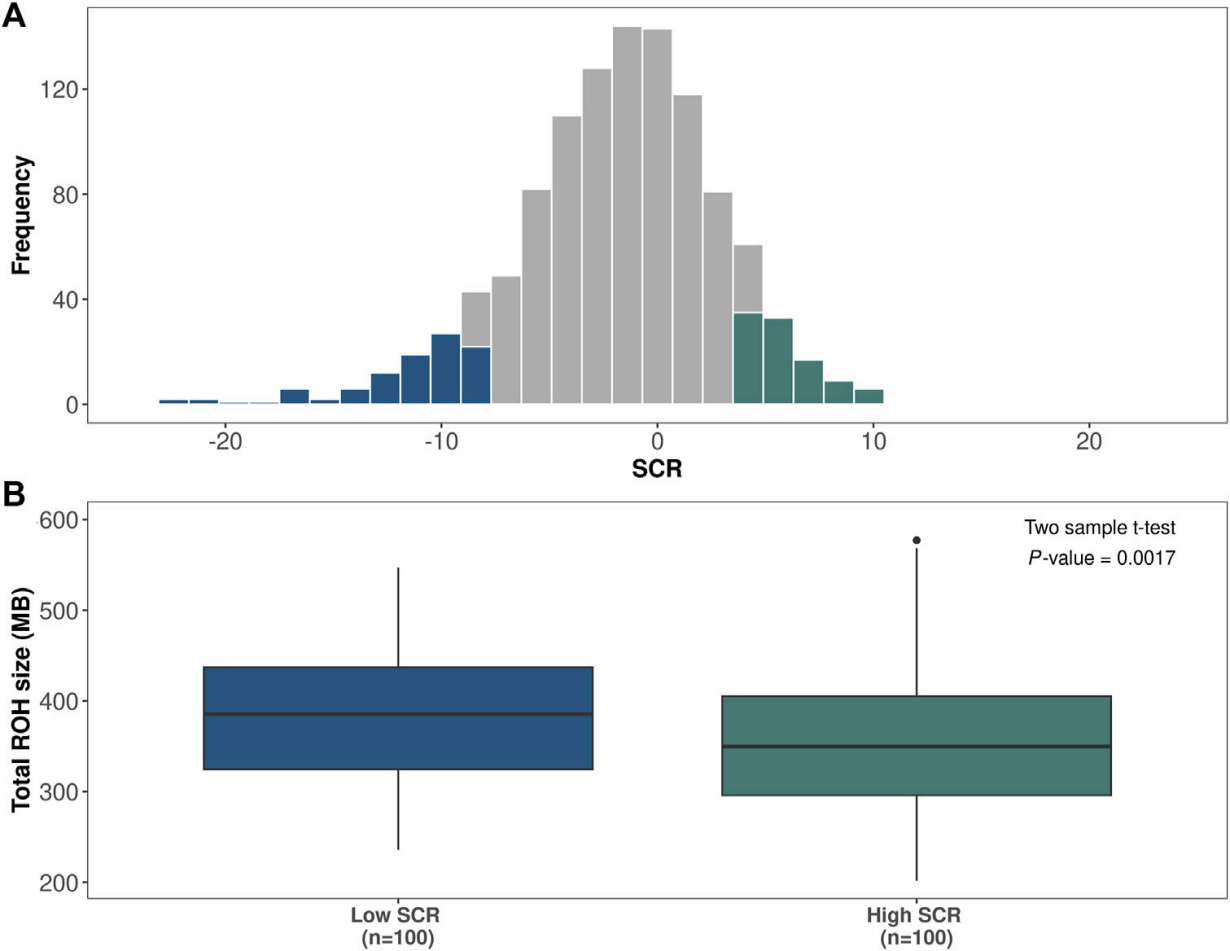
Association between runs of homozygosity (ROH) and bull fertility. **(A)** Histogram showing the distribution of sire conception rate (SCR) records in the Italian Brown Swiss bull population (*n* = 1,102). The bottom 100 low-fertility bulls and the top 100 high-fertility bulls are highlighted in blue and green, respectively. **(B)** Distribution of amount of homozygosity, calculated as total ROH length, for low-fertility (*n* = 100) versus high-fertility (*n* = 100) bulls.

Figure 3A shows the distribution of SCR values for the Italian Brown Swiss population; the bottom 100 bulls and top 100 bulls of the distribution are highlighted. The total homozygosity length was measured as the sum of all ROH segments for each animal, and significant differences (*p*-value ≤0.01) were found between the two fertility groups. Indeed, the total ROH length average was equal to 387 Mb for the low-fertility bulls and 353 Mb for the high-fertility bulls (Figure 3B).

### 3.3 Enrichment of ROH in low-fertility bulls

**FIGURE 4:**
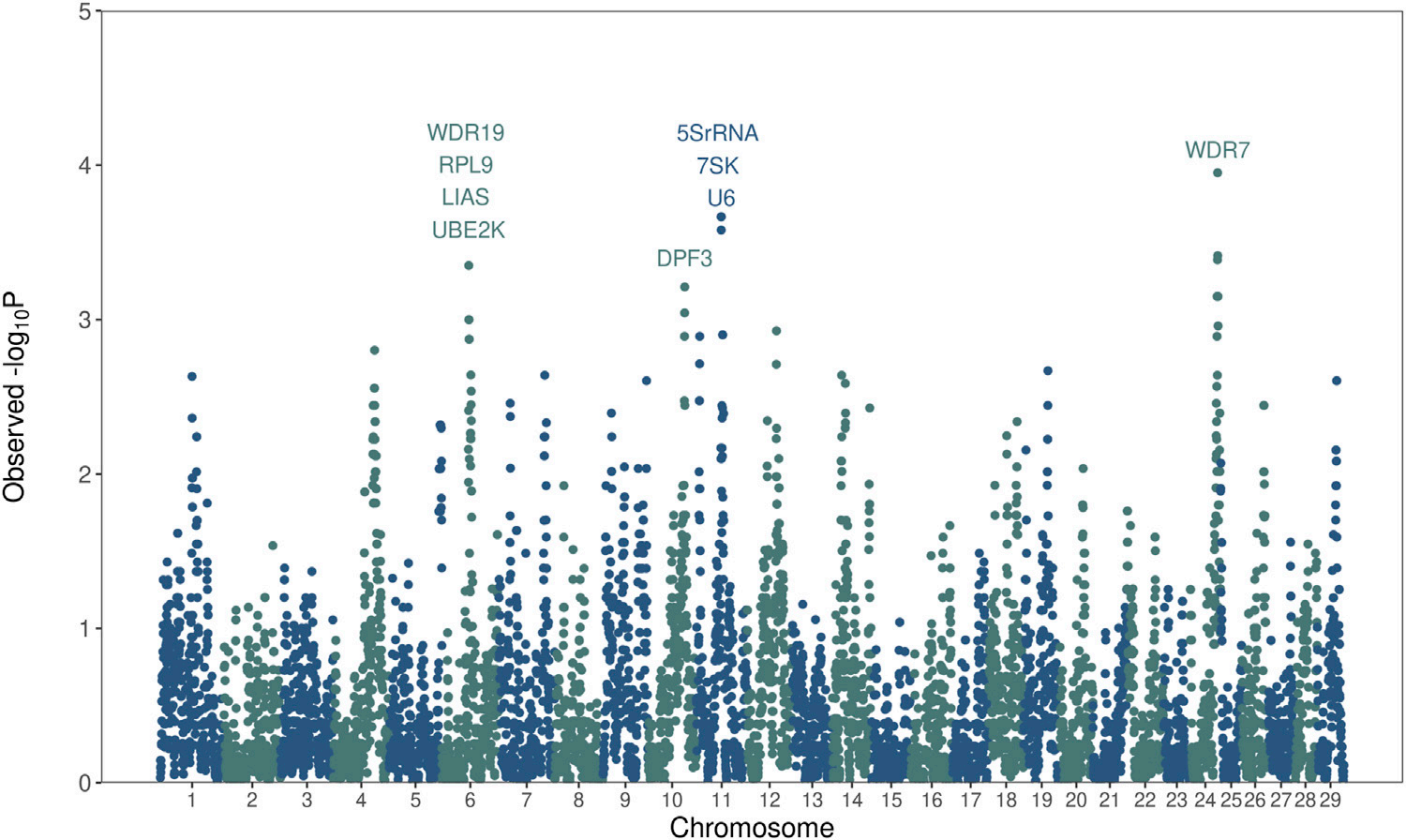
Whole-genome homozygosity mapping. The enrichment of runs of homozygosity (ROH) in low-fertility bulls evaluated using a Fisher’s exact test. The Manhattan plot shows the significance of each ROH region (*y*-axis) across the entire autosome genome (*x*-axis). Candidate genes affecting bull fertility are annotated.

A total of 10,282 overlapping ROH regions were detected across the entire autosomal genome (Supplementary Table S1). The mean length of overlapping ROH was 57.4 kb, with an average of 14 SNPs. The longest overlapping region was 1,117 kb, and the maximum number of SNPs was 225 SNP. The enrichment of overlapping ROH segments in bulls with low fertility was evaluated using a one-tailed Fisher’s exact test. Twenty-two overlapping ROH segments located in chromosomes 6, 10, 11, and 24 were found to be significantly enriched in low-fertility bulls (Figure 4; Supplementary Table S2). Remarkably, these genomic regions harbor genes such as *WDR19*, *RPL9*, *LIAS*, *UBE2K*, *DPF3*, *5S-rRNA*, *7SK*, *U6*, and *WDR7* that are closely related to sperm biology and male fertility.

### 3.4 Validation of significant ROH regions

For the validation process, we only considered the four most significant ROH segments identified in the enrichment analysis. The significant association between these four ROH segments and male fertility was validated using the entire Italian Brown Swiss bull population. Notably, all four overlapping ROH regions were significantly associated with sire conception rate records in the entire population (|t-value| ≥ 2). The position of the ROH segments along with *p*-values (Fisher’s exact test) and *t*-values (validation analysis) are listed in Table 1.

**TABLE 1:** Homozygous regions significantly enriched in low-fertility Italian Brown Swiss bulls (*n* = 100) and validated in the entire population (*n* = 1,102).

Chromosome	Start (bp)	End (bp)	*p*-value	*t*-value
6	57,710,747	57,922,701	4.46 × 10^−4^	−3.98
10	84,137,534	84,185,741	9.05 × 10^−4^	−3.29
11	51,245,756	51,289,911	2.16 × 10^−4^	−3.45
24	55,073,708	55,139,885	7.08 × 10^−4^	−3.14

## 4 Discussion

### 4.1 Discovery and comparison of ROH between low- and high-fertility bulls

The average length of the ROH (4.6 Mb) was similar to what was previously reported in other studies using Brown Swiss animals (Ferencakovic et al., 2013; Marras et al., 2015; Cesarani et al., 2018). The average ROH segments per animal, *n* ≈ 79, was lower than what was found by Ferencakovic et al. (2013) and Marras et al. (2015), that is, *n* ≈ 99 and *n* ≈ 94, respectively, possibly because we used a minimum threshold of 100 SNPs rather than the 15 SNPs. Interestingly, Brown Swiss cattle consistently have the largest average ROH length when compared to other dairy breeds (Nani and Peñagaricano, 2020; Lozada-Soto et al., 2022). This may be attributed to intense selection and a small effective population size, resulting in a high frequency of long homozygous segments across the genome. Indeed, Kim et al. (2013) had reported that selection within a dairy cattle population leads to an overall increase in autozygosity throughout the genome, while autozygosity in an unselected line remains relatively stable across most of the chromosomes. This shows that the variations in selection history among different dairy cattle populations lead to diverse ROH patterns observed across breeds.

The distribution of total homozygosity among chromosomes was relatively even but higher in chromosomes 5 and 6, which was as shown by Marras et al. (2015). The distribution of ROH is correlated not only with genomic features such as GC content but also with selection and recombination rates, as longer ROH tend to occur more frequently in areas of the genome with lower recombination (Kirin et al., 2010; Bosse et al., 2012). Interestingly, there is no evidence of a connection between chromosome size and the amount of homozygosity. One possible reason for the higher homozygosity in chromosomes 5 and 6 is the selection for milk production traits. Indeed, Lozada-Soto et al. (2022) showed that BTA5 and BTA6 are enriched in traits related to milk production, such as milk yield, milk component, and lactation persistency. Likewise, Kim et al. (2013) had demonstrated that the distribution of ROH varies more extensively across the genomes of selected animals, with several regions exhibiting higher homozygosity levels. This supports the notion that artificial selection can impact ROH frequency and size.

The pedigree-based inbreeding coefficients were moderately correlated with the ROH values (0.65). They were consistently lower than the ROH estimates. Marras et al. (2015) had obtained similar results when comparing both methods using Brown Swiss animals. It is important to note that ROH captures both historical and recent relatedness through shorter and longer IBD fragments, respectively. On the other hand, pedigree-based inbreeding provides estimates solely based on pedigree data that may not expand beyond a few generations, resulting in an underestimation of inbreeding coefficients.

We evaluated the impact of homozygosity on service sire fertility by assessing the association between ROH and SCR values. Our findings indicate that bulls with longer homozygous regions and/or a greater number of homozygous segments in their genome exhibit lower male fertility. Interestingly, Nani and Peñagaricano (2020) had also found a negative association between ROH and the sire conception rate in US Holstein bulls. Previous studies have evaluated the impact of homozygosity on sperm biology, showing that different ROH regions affect the total number of spermatozoa and the percentage of live spermatozoa in Austrian Fleckvieh bulls (Ferencakovic et al., 2017). Similarly, negative effects of inbreeding were reported for semen traits in Holstein bulls (Ghoreishifar et al., 2023).

We also investigated the differences in homozygosity between low- and high-fertility animals, the bottom 100 and top 100 Brown Swiss bulls. Notably, there was a significant difference in the levels of total homozygosity between the two fertility groups, measured as the sum of ROH. Moreover, 22 overlapping ROH segments in four different chromosomes (BTA6, BTA10, BTA11, and BTA24) were significantly enriched in low-fertility bulls. Statistical validation of the four most significant ROH segments per chromosome was conducted on the entire population, indicating that these findings are not false-positives resulting from the population structure. Remarkably, the region identified in BTA6 had been previously reported as a region associated with bull fertility in Brown Swiss cattle (Hiltpold et al., 2020; Hiltpold et al., 2021; Mapel et al., 2022; Pacheco et al., 2022). These results suggest that homozygosity might be an important adverse factor for male fertility in cattle.

### 4.2 ROH significantly enriched in low-fertility bulls

The significant ROH segments in BTA6 contain several candidate genes, such as *WDR19*, *RPL9*, *LIAS*, and *UBE2K*. The gene *WDR19* is a strong candidate for service sire fertility in Brown Swiss cattle. The gene *WDR19* is a constituent of the intraflagellar transport complex essential for the physiological functioning of motile cilia and flagella, such as in sperm motility (Ni et al., 2020). Interestingly, previous studies have identified a variant in *WDR19* that is significantly associated with various semen traits, such as sperm motility and sperm abnormalities, insemination success, and SCR, in Brown Swiss cattle (Hiltpold et al., 2020; Hiltpold et al., 2021; Mapel et al., 2022; Pacheco et al., 2022). The candidate gene *RPL9* encodes a ribosomal protein, which belongs to the L6P family of ribosomal proteins. The gene *RPL9* has been identified as differentially expressed in idiopathic male infertility patients, suggesting that it may play a vital role in male fertility (Lukkani et al., 2022). The gene *LIAS* is a lipoic acid synthase gene and predominantly expressed in the testes. Recent research suggests that *LIAS* may play a critical role in spermatogenesis, as it was shown to be downregulated in cases of spermatogenic dysfunction in humans (Zhao et al., 2023). The *UBE2K* gene encodes a protein that is a member of the ubiquitin-conjugating enzyme family and is crucial for the ubiquitination pathways, which are essential to the later stages of spermatid development during spermiogenesis (Anbazhagan et al., 2023). Malfunctions in the ubiquitination system can impair sperm development, ultimately resulting in male infertility (Nakamura, 2013). It is noteworthy that these four candidate genes identified on BTA6 exhibit medium to high levels of expression in the testes in Brown Swiss bulls (Hiltpold et al., 2020).

The region in BTA10 harbors *DPF3*, a potential candidate gene related to bull fertility. This gene encodes a member of the D4 protein family of zinc finger proteins involved in histone modification. Studies have suggested that *DPF3* may play a role in spermatogenesis through chromatin remodeling, as epigenetic factors such as histone modifications can potentially affect spermatogenesis and embryogenesis. Notably, variants within *DPF3* have been strongly associated with male infertility and sperm morphology in diverse ethnic groups from the US and China (Kosova et al., 2014; Liu et al., 2017; Sato et al., 2018).

The region in BTA11 contains three non-coding RNAs: 5S-rRNA, 7SK, and U6. The 5S-rRNA, a ribosomal RNA, is part of a sperm-specific DNA organization that may be involved in specific patterns of DNA replication and transcription of the paternal genome in the embryo (Nadel et al., 1995). Borowska et al. (2018) reported that several SNPs associated with sperm traits in Holstein bulls were in 5S-rRNA. Moreover, RNA 7SK has a role in controlling primordial germ cell proliferation in mouse embryos (Okamura et al., 2012). Notably, 7SK was detected in seminal plasma, ejaculated sperm, and epididymal sperm in pigs, suggesting that this RNA has a significant biological role in spermatogenesis (Chen et al., 2017). In Holstein bulls, some SNPs associated with sperm traits were in U6, a small nuclear RNA (Suchocki and Szyda, 2015; Borowska et al., 2018). Hence, non-coding RNAs could be promising biomarkers for assessing sperm quality (Hua et al., 2019). Finally, the significant ROH segment in BTA24 harbors the gene *WDR7*, which is significantly associated with semen production traits in Holstein bulls (Suchocki and Szyda, 2015).

### 4.3 Applications of ROH

The assessment of ROH is a powerful tool for understanding population history, assessing genomic inbreeding, and identifying genes linked with critical economic traits. It can help minimize inbreeding rates and prevent the loss of genetic diversity in dairy cattle. The quantification of ROH and identification of homozygous regions might contribute to the design of mating programs aimed at minimizing inbreeding and avoiding the production of homozygous offspring, which may carry deleterious alleles for male fertility. Moreover, the association between ROH and dairy bull fertility helps us understand the impact of intense artificial selection on the trait and prevent even more inbreeding and the loss of genetic diversity in the dairy cattle population. In this study, we found a higher prevalence of ROH in low-fertility Italian Brown Swiss bulls than in high-fertility bulls, indicating that inbreeding and increased homozygosity negatively affect dairy bull fertility. Conducting genome-wide mapping of ROH enables the identification of potential genes affecting bull fertility, leading to a deeper understanding of the molecular mechanisms underlying male fertility in cattle. It is important to acknowledge that our bull fertility data set is based on cow field records and is subject to pre-selection, as only bulls meeting specific sperm quantity and quality criteria are included.

## 5 Conclusion

In this study, we evaluated the presence of ROH in Italian Brown Swiss dairy cattle and assessed its association with bull fertility. Remarkably, there was a negative association between bull fertility and the amount of homozygosity. We observed a significant difference in the levels of total homozygosity between the low- and high-fertility groups. Interestingly, the ROH segments enriched in low-fertility bulls harbor genes that are directly involved in sperm biology and male fertility. Our findings suggest that inbreeding and increased homozygosity harm male fertility in Brown Swiss cattle. The quantification of ROH can be used to minimize inbreeding and avoid its negative effect on fitness-related traits, such as bull fertility.

## Data Availability

The data analyzed in this study is subject to the following licenses/restrictions: the phenotypic and genotypic data analyzed in this study were obtained from the Italian Brown Breeders Association. These data sets were used under agreement and are hence not publicly available. However, data are available upon request to FP and with the permission of the Italian Brown Breeders Association. Requests to access these data sets should be directed to FP, fpenagarican@wisc.edu.
